# Anorectal Malformation: Paediatric Problem Presenting in Adult

**DOI:** 10.1155/2015/625474

**Published:** 2015-10-11

**Authors:** Rahulkumar N. Chavan, Bhargav Chikkala, Cinjini Das, Somak Biswas, Diptendra Kumar Sarkar, Sushil Kumar Pandey

**Affiliations:** ^1^PGT, Department of General Surgery, IPGMER, Kolkata, India; ^2^DY Patil University School of Medicine, Maharashtra, India; ^3^PGT, IPGMER, Kolkata, India; ^4^TMH, Kolkata, India; ^5^KEM Hospital, Mumbai, India; ^6^Department of General Surgery, IPGMER, Kolkata, India; ^7^RMO, IPGMER, Kolkata, India

## Abstract

This is a case report of 22-year-old girl admitted with abdominal distension, vomiting, and chronic constipation since birth. Abdomen was distended, and perineal examination revealed imperforate anus with vestibular fistula (ARM). So far worldwide very few cases have been reported about anorectal malformation presenting in adulthood, and thus extremely little data is available in the literature about an ideal management of anorectal malformation in adults. In our case in the treatment instead of conventional procedure of posterior sagittal anorectoplasty (PSARP) anal transposition was done and till two years after the definitive treatment during follow-up patient has been doing well with Kelly's score of six. Our experience suggests that anal transposition provides satisfactory outcome in adults presenting late with anorectal malformation.

## 1. Introduction

Though anorectal malformation (ARM) is pediatric problem, in India because of poverty and ignorance one may encounter it in adults also. Posterior sagittal anorectoplasty (PSARP) is now accepted as a standard treatment in children, but there is very little literature guiding an ideal treatment of anorectal malformation in adults. Here we present a case of an adult female who was neglected since childhood and presented with congenital low type of anorectal malformation, we performed anal transposition which gave her a good outcome.

## 2. Case History

**Figure 1:**
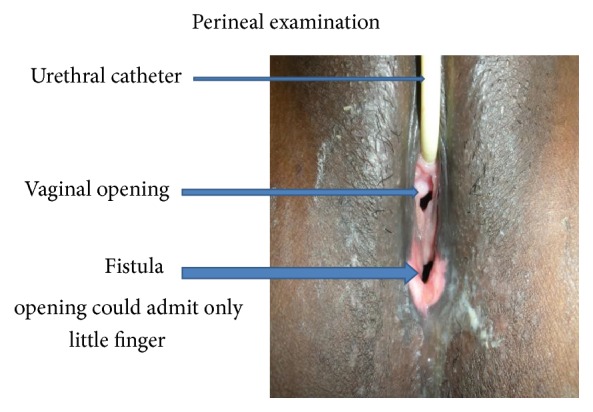
Showing anovestibular fistula in an adult female patient with openings of urethra and vagina. Note no separate anal opening.

22-year-old female presented with features suggestive of large bowel obstruction, chronic constipation since birth and regular passage of small quantity of stool through an opening in the perineum. Perineal examination revealed no separate anal opening (Figure 1), dry stool seen through an opening in vestibule.

Transverse diameter inside the fistulous opening appeared very large on P/R exam and stool gave a firm feel to finger. X-ray abdomen showed loaded colon. She had no other congenital anomalies. In the first setting after primary resuscitation an emergency laparotomy was carried out and proximal diverting transverse loop colostomy was done to relieve the obstruction. After this under anesthesia stepwise instrumental evacuation of stool was performed through fistulous opening as hard and enormous quantity did not yield to conservative treatment, through either stoma or distal fistula. Few days later distal cologram showed decompressed distal colon and finally patient was planned for definitive procedure.

### 2.1. Intraoperative Details

**Figure 2:**
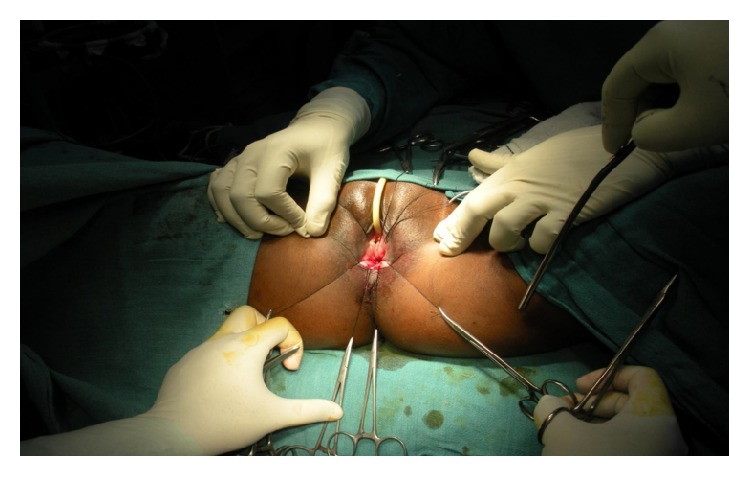
Exposure of operative area.

**Figure 3:**
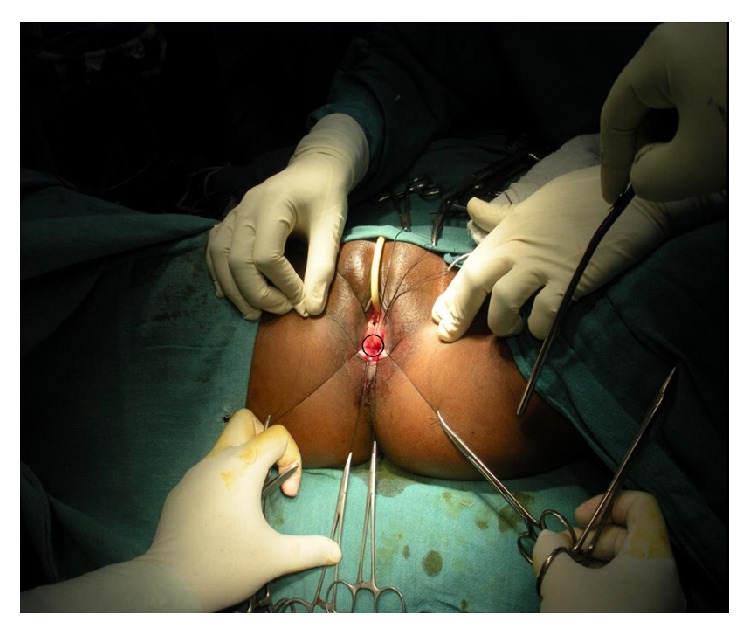
Line of incision depicted around the anorectum.

**Figure 4:**
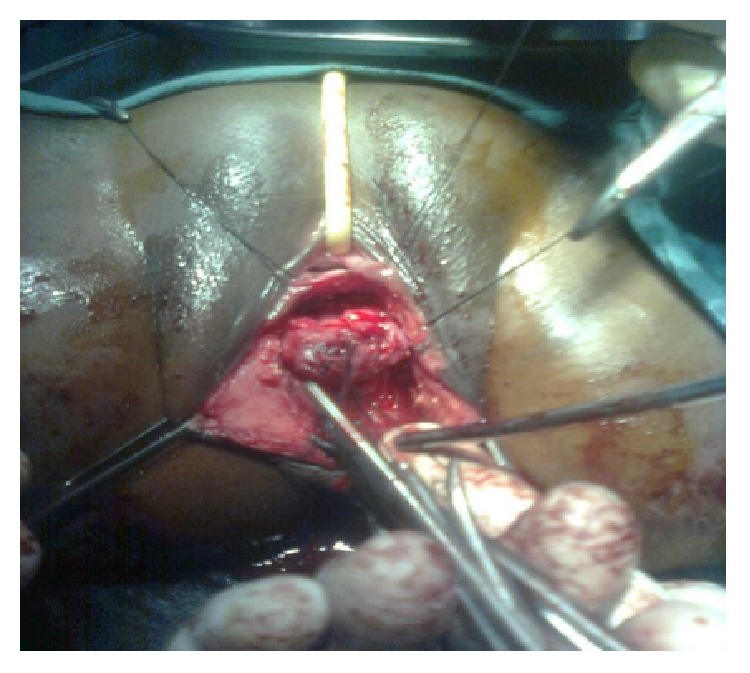
Circumferential mobilization of anorectum.

**Figure 5:**
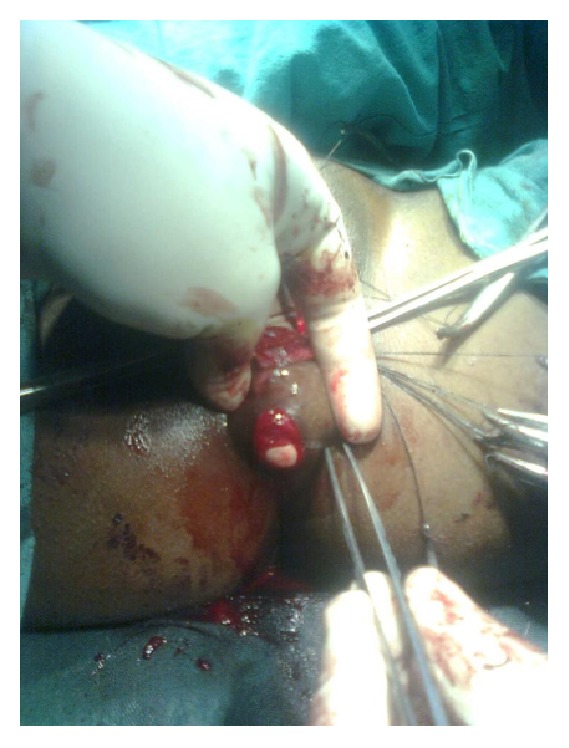
New anal opening created.

**Figure 6:**
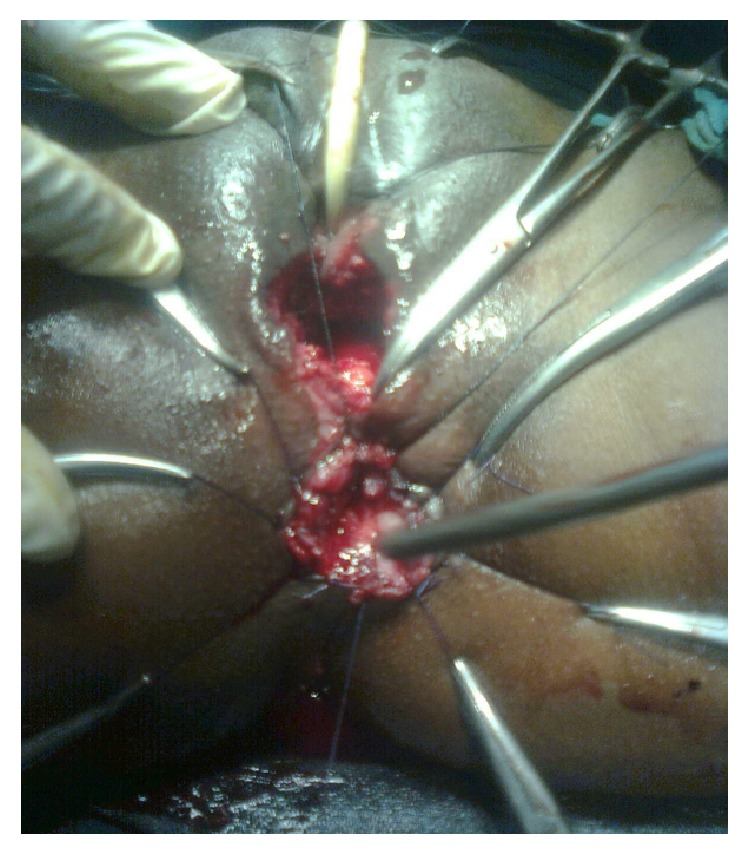
Anorectum transposed within sphincteric complex.

Under spinal anaesthesia patient was put in lithotomy position, thorough examination was done, and previous clinical findings were confirmed. Tractions sutures applied to expose the operative area (Figure 2), Inj. Adrenalin with concentration of 1/1000 in saline was injected around the fistula to help in dissection. Circumferential incision was made around fistulous opening, as depicted in Figure 3. Posterior wall of vagina separated from the anterior wall of rectum anorectum is circumferentially mobilized up to the level of levator muscle (Figure 4). With the help of muscle stimulator sphincter complex was identified in the perineum and an opening made within it (Figure 5); electrocautery use minimized at this stage to prevent injury to sphincter complex (Figure 2). Tunnel was created through sphincteric complex and mobilised anorectum pulled down through it, fixed at the new opening within; thus anal transposition was done (Figures 5 and 6). There was a small rent in vaginal wall which was repaired with absorbable suture.

**Figure 7:**
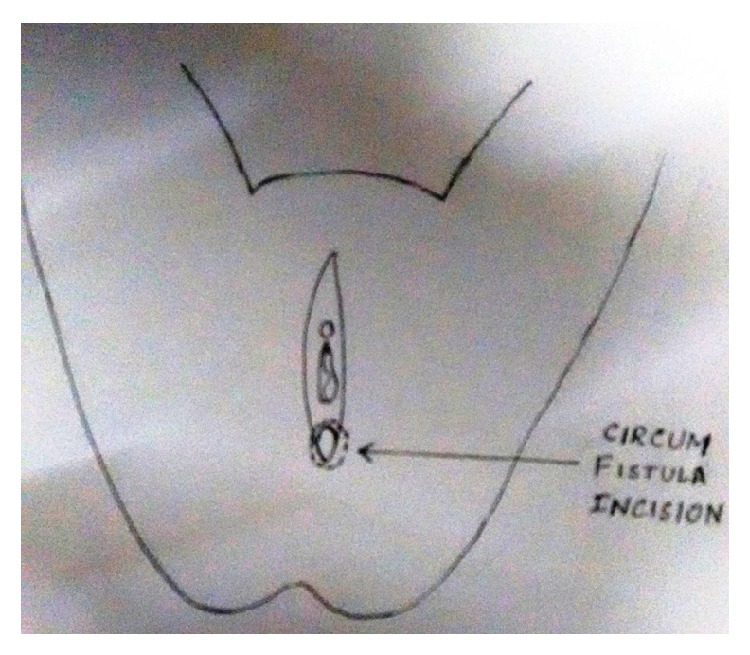
Incision around the fistulous opening.

**Figure 8:**
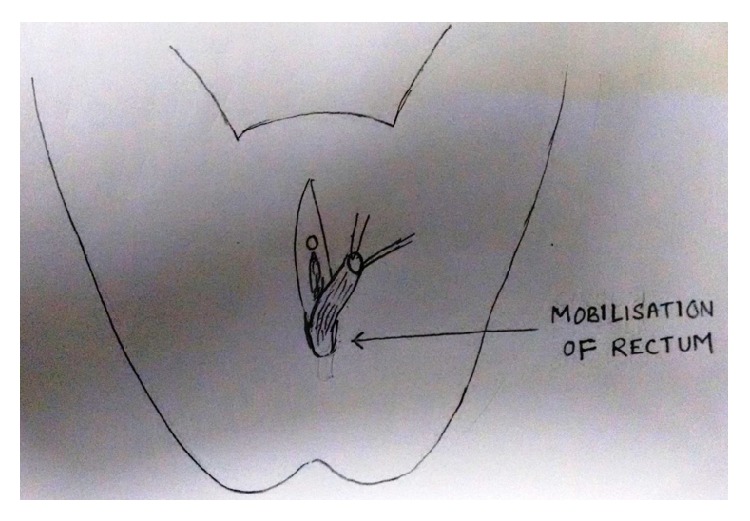
Mobilisation of rectum.

**Figure 9:**
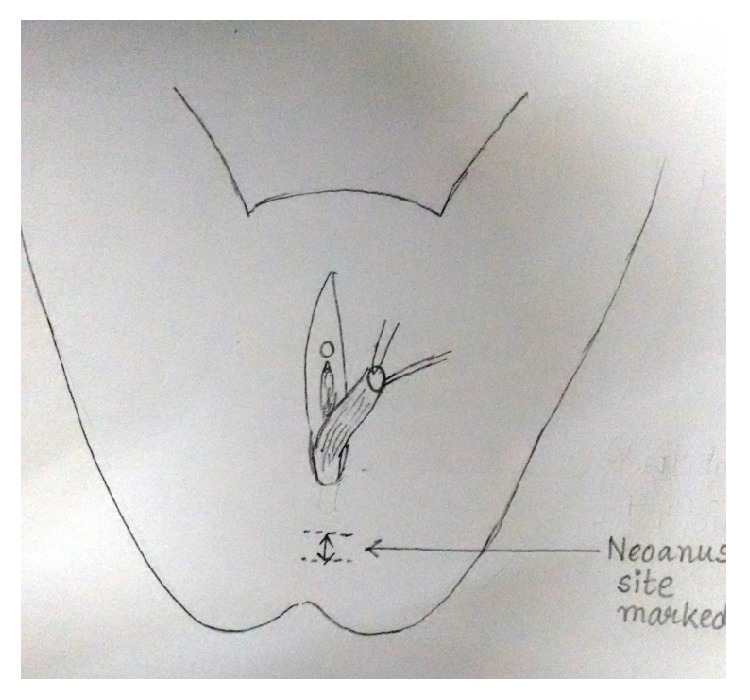
New anal opening marked with the help of muscle stimulator.

**Figure 10:**
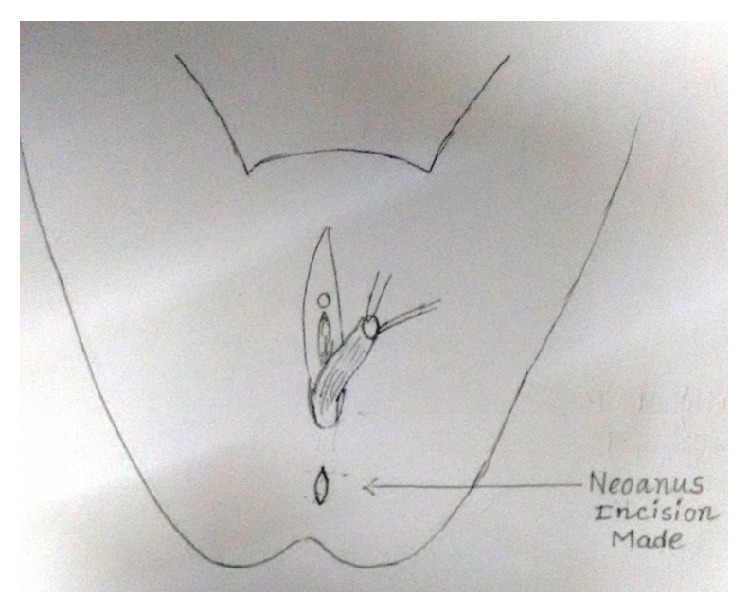
Incision made at proposed new anal opening.

**Figure 11:**
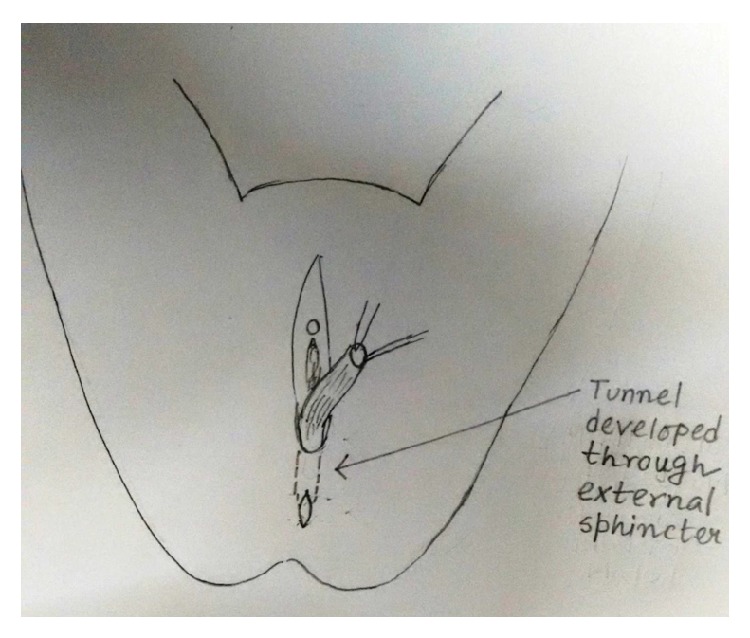
Tunnel developed for mobilisation of rectum.

**Figure 12:**
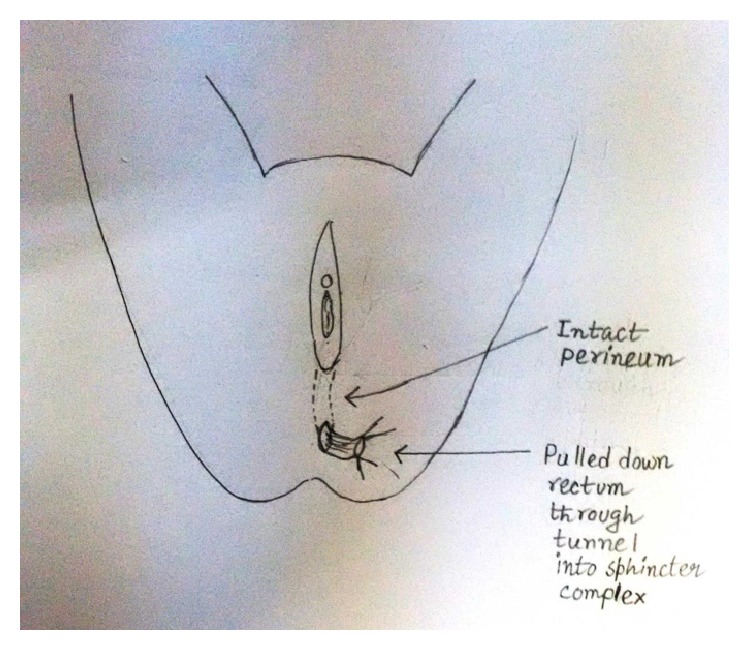
Rectum transposed.

**Figure 13:**
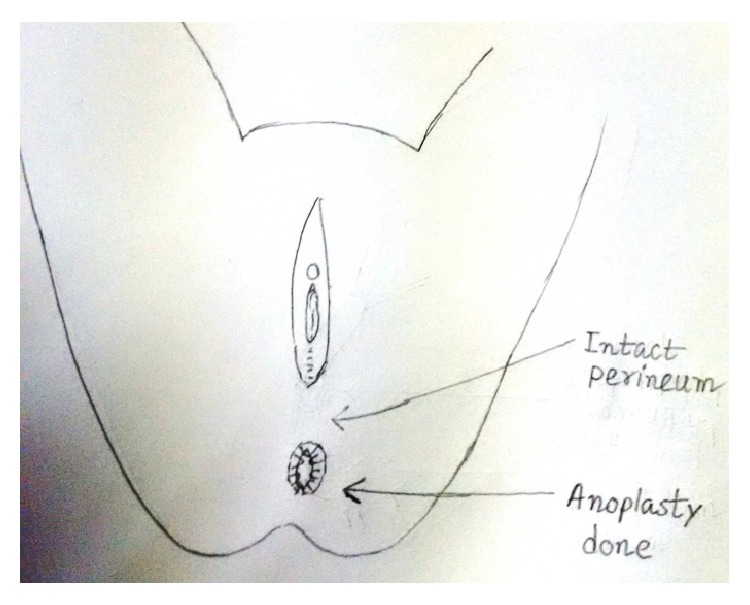
Anoplasty completed.

Diagrammatic representation of the procedure has been shown sequentially in Figures 7, 8, 9, 10, 11, 12, and 13. 

### 2.2. Postoperative Course

Skin sutures were removed after 12 days and 2 weeks after the procedure sequential anal dilatation started, patient was continent. For 2 months along with anal dilator she was maintained on stool softener and high fibre diet. After 2 months stool softener use was tapered. Three months later distal loop cologram showed patent passage, so temporary colostomy was closed. Her anal manometry study was satisfactory. She had voluntary defecation without soling or constipation. She was advised to strictly follow dietary modification onwards. We used Kelly's score to assess her physiologic function during follow-up. At the end of 1 year she was continent without soiling or constipation. She could differentiate between feces and flatus and had strong effective squeeze, with Kelly's overall score of six. Though no objective evaluation was done, she was satisfied with perineal cosmetic outcome.

## 3. Discussion

The most common anorectal malformation defect in females is imperforate anus with vestibular fistula. In females this anovestibular fistula is a low type of disease and it opens near the vagina at the posterior fourchette and is directed posteriorly and upward with adhesion to posterior vaginal wall [1, 2]. In ano/rectovestibular fistula rectal pouch has already passed through the levator ani muscle so mobilisation of the rectal pouch can be done without cutting the muscle. Contrary to children adult patients do have potential of bowel control so retaining a fecal continence and maintaining integrity of sphincter complex should be the goal in correction of anorectal malformation in adults. Various surgical approaches to treat ARM have been suggested and these are like PSARP (posterior sagittal anorectoplasty), ASARP (anterior sagittal anorectoplasty), and TSARP (transsphincter anorectoplasty which is also called anal transposition) [3]. In cases of low lesion in children primary perineal repair can be performed without need for the stoma, but as we experienced in present case, in adults who have chronic constipation, large quantity of dry stool may not allow single stage definitive procedure. Though PSARP suggested by Pena et al. continues to be the treatment of choice for ARM in children, in adult due to extreme rarity of incidence there is no recommended treatment and possibility of PSARP is yet uncertain [4]. There are isolated case reports of anorectal malformation in adults treated successfully with PSARP [4, 5] but this approach involves surgically dividing the puborectalis component of levator muscles and muscle complex (which play a very important role in the continence mechanisms [6]), perineal body, and the perineal skin. This can cause wound complications like scar of the perineal skin bridge between the fistula and the new anus [3]. This complication can be avoided with anal transposition which we performed in our case, which retains the integrity muscle complex, the perineal body, and the perineal skin [7]. Constipation though expected to be more common in the anal transposition can be managed with lifestyle changes. Anal transposition can also be called TSARP (transsphincter anorectoplasty) [3]. There are many scoring systems to evaluate outcome of anorectal malformation repair. Here we used Kelly's score [8], and we found it is very useful for assessment of anorectal physiology after anal transposition even in adults.

## 4. Conclusion

We feel anal transposition provides satisfactory results in adult patients and also those presenting with anorectal malformation, it provides clear recognition of sphincteric complex with good cosmetic and functional outcome, and result is comparable with PSARP done for children.
